# P-1260. Validation of Modified First-Order Kinetic Equation for Vancomycin Area Under the Curve Calculation in Neonatal Patients

**DOI:** 10.1093/ofid/ofae631.1442

**Published:** 2025-01-29

**Authors:** Tsung-Chi Lien, She-Yi Wei, Harlan Husted, Sahar Taha, Alice Ip, Laurie Covarrubias, Chokechai Rongkavilit

**Affiliations:** Community Regional Medical Center, Fresno, California; Valley Children's Hospital, Fresno, California; Community Regional Medical Center, Fresno, California; Community Regional Medical Center, Fresno, California; Community Regional Medical Center, Fresno, California; Community Regional Medical Center, Fresno, California; University of California San Francisco, Fresno Branch Campus, Fresno, California

## Abstract

**Background:**

Current guideline emphasizes a ratio of 24 hour-area under the curve (AUC) to minimum inhibitory concentration (MIC) of ≥ 400 mg.h/L as the primary pharmacokinetic and pharmacodynamics predictor of vancomycin activity against serious methicillin-resistant *S. aureus* (MRSA) infections. Premature infants admitted to neonatal intensive care unit (NICU) are at high risk of Staphylococcal infections due to immunocompromised state and frequent procedures; however, the availability of vancomycin AUC calculator for this population is limited.

**Methods:**

Neonates received vancomycin for at least 4 days between January 2018 and December 2023 with steady-state trough level were included. The modified first-order kinetic equation using population-based distributive volume (Vd_f_) of 0.7 L/kg and trough level were compared to the Vanc App program validated in infants aged 0 to 90 days with post-menstrual age (PMA) ≥ 25 weeks and weight ≥ 500 grams. Ratio and percentage difference of calculated AUCs between the two models were assessed, as were safety parameters, including urine output < 0.5 mL/kg/hr over at least 6 hours, serum creatinine increases by 0.3 mg/dL or 1.5 times baseline, duration of culture positivity, and recurrence of same infection within 14 days.

**Results:**

We identified 95 neonates with 56% male. The median (range) PMA was 30.1 (24.2 - 44.3) weeks, age was 21 (1 - 137) days, and weight was 1.2 (0.54 - 4.30) kg. Five cases had culture-proven MRSA infections. The average vancomycin dose was 37 mg/kg/day with median (range) duration of 7 (4 - 45) days. Acute kidney injury was identified in 2.1% of patients. Target AUC was achieved in 78% of cases. The average duration of culture positivity was 2.2 days, and no patients exhibited recurrent infections within 14 days after stopping vancomycin. The median (IQR) accuracy was 0.98 (0.95 - 1.01), and bias was -0.02 (-0.05 - 0.01). To obtain concordant AUC results, the mean (SD) Vd_f_ was 0.67 (0.09) L/kg. No statistically significant differences between the two models in identifying AUC outside of therapeutic range (p=0.083).

**Conclusion:**

The modified first-order kinetic equations can achieve comparable AUC estimates in neonates by incorporating a population PK-based VD_f_ of 0.7 and single trough level, eliminating the need for additional blood draws.

**Disclosures:**

**All Authors**: No reported disclosures

